# Annotations

**Published:** 1901-07-13

**Authors:** 


### Annotations.

### Street Cries: An Education in Law Breaking.

London is an exceptional place, and perhaps it is right that it should receive exceptional treatment. Being the capital of a great empire it may be proper that its police should be directly controlled by the Government rather than by any local body. All the same the arrangement has its inconveniences. In any provincial town if the corporation were to make a by-law for the good conduct of its streets, we may be quite sure that the police who are employed by the corporation would have to see that this by-law was enforced. In London, however, things are quite different. Some time ago the London County Council saw fit to pass a by-law with the object of suppressing a public nuisance of considerable magnitude, namely, the shouting in the streets which is indulged in by newspaper hawkers to the annoyance of all decent inhabitants, and greatly to the injury of brain workers and of all sick folk. But the whole thing is a dead letter, and the shouting goes on as badly as ever. Such a condition of affairs, such a public toleration and encouragement of law-breaking, would not be permitted for a moment in a provincial town. It may be necessary for great reasons of State that the police of London should be at the disposal of the Home Office, but if so some arrangement should be come to by which they could be made use of for enforcing the by-laws of the local authorities, or else these authorities should be allowed to employ a police force of their own for local purposes. The only other alternative would be that the local authority should no longer be allowed to pass by-laws. In any case something should be done to put a stop to the education in law-breaking which is now going on. We have a beautiful organisation which theoretically commits to the various municipal bodies the care of their several districts, and for the proper governance of these districts they may pass all sorts of useful by-laws, as also may that larger body, the County Council, in regard to matters properly coming before it. Many of these by-laws are likely to be of the highest importance from a sanitary point of view. But whether they will ever be enforced depends upon the view taken of them by the Home Office and the Chief Commissioner of Police, unless, indeed, some self-sacrificing private citizen can be found who will himself make complaint—a proceeding which in view of the present organisation of our police courts is hardly to be expected. So we find people on every hand doing with impunity what they know is contrary to law, and so is law brought into general contempt.

### Incipient Lunacy.

We are constantly receiving letters asking what is to be done with cases which although said to be "mentally affected," are yet not considered fit for asylum treatment. And it is by no means an easy question to answer. The law draws no distinctions. Lunatics and persons of unsound mind are all classed together, and only at his peril and at the risk of many penalties, may a man take charge of any such persons, except under a certificate. Yet many of these people are uncertifiable. The very essence of the certificate is that the patient is a "proper person to be taken charge of and detained" under care and treatment. But there are scores of persons of unsound mind in regard to whom this cannot properly be said, and it always seems to us a great hardship that difficulties should be placed in the way of people of this kind even receiving ordinary board and lodging, not to mention care and treatment. "Every person who, except under the provisions of this Act (that is under certificate) . . . for payment takes charge of, receives to board or lodge, or detains, a lunatic or alleged lunatic in an unlicensed house shall be guilty of a misdemeanour and shall also be liable to a penalty not exceeding £50." What then is to be done for the poor wretch who is not sound in mind and yet not fit for bolts and bars? It is a cruel difficulty which urgently demands rectification, for be it remembered it is just at this time when the patient's mind is only beginning to show signs of derangement that a complete change of surroundings is most desirable, and that under the influence of such change recovery may be hoped for. The law, however, stands in the way, making the reception of such a person for payment a misdemeanour, and although we are well aware that this law is evaded, and that the "slight mental case" received without certificate, often gets well without anyone hearing about it, the fact remains that such reception is contrary to law, and that in consequence the most able and conscientious men—the very men to whom one would most willingly entrust the care of those who are on the borderland—are warned off from undertaking this most useful work. We cannot but feel that if a man gave notice to the Commissioners that he had such a case under his care that ought to be enough, and that some modification of the law in that sense ought speedily to be made, so that instead of incipient lunacy drifting on until it reaches a condition at which it is certifiable, it shall come under proper care at its earliest and therefore its most amenable stage.

### The Expert Committee on the Army Medical Service.

Some surprise has been expressed at the small representation given to the Royal Army Medical Corps in the committee of experts which has been appointed to assist in the elaboration of a scheme for the reorganisation of the Army Medical Service. It may be well, however, to point out that the committee was not appointed to undertake the formulation of a scheme, but to consider a scheme which had been drawn up by Mr. Brodrick, presumably with the assistance of those in office at the Medical Department, which is a very different matter. From the constitution of the committee we can have but little doubt that the intention is to obtain an opinion as to the manner in which the scheme will appeal to the profession outside, and how far it will meet the wants of the service as they have shown themselves to those who, from the outside rather than from within, have watched the working of the present system in the field. It is to be observed that the committee includes not only some who have spoken pleasant things of the Royal Army Medical Corps, but one at least who even before the outbreak of this war had criticised in the strongest terms not only the system but the officers whom it produced. A great responsibility attaches to those who serve on this committee, for whether they like it or not, there can be no doubt that they will have to share, and to a large extent to bear, the responsibility for any scheme which may ultimately be propounded.

